# Effects of Supramolecular Interactions on *E/Z*‐Isomerizations in Water and Their Importance for Biological Applications

**DOI:** 10.1002/ardp.70242

**Published:** 2026-04-21

**Authors:** Doğan Akbulut, Gianna Pölderl, Line Næsborg

**Affiliations:** ^1^ Organisch‐Chemisches Institut Universität Münster Münster Germany

**Keywords:** aqueous environment, biomimetic systems, *E*/*Z*‐isomerization, photochemical control, supramolecular interactions

## Abstract

Supramolecular systems in aqueous media function as biomimetic reaction environments, enabling the investigation of microenvironmental effects and molecular interactions. Photochemical reactions in these complex media facilitate the development of photochemical tools for biological research. It is showcased herein how *E*/*Z*‐isomerizations are influenced by supramolecular interactions. Afterwards, the importance of *E*/*Z*‐isomerizations for biological applications is highlighted, for example, in the context of ion channels. These examples demonstrate that interactions between supramolecular entities and small molecules undergoing *E*/*Z*‐isomerizations significantly influence the observed experimental outcomes.

## Graphical abstract

Biomimetic supramolecular hosts in water affect outcomes and behavior of *E*/*Z*‐isomerizations. A deeper understanding of such effects can pave the way to understanding photochemical processes in more complex biological environments. The review displays examples of supramolecular hosts affecting the outcomes of *E*/*Z*‐isomerizations and subsequently highlights relevant biological applications.

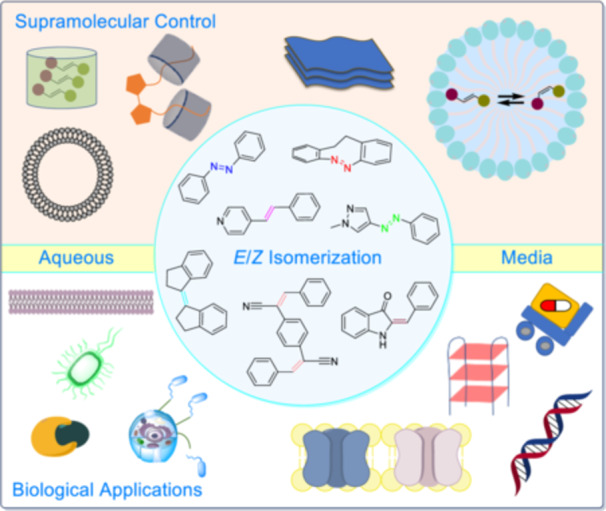

## 1 Introduction

The local environment can significantly influence the outcome of chemical reactions. Supramolecular entities are able to introduce changes in the local environment surrounding the substrate. Consequently, changes in the local environment will affect photoinduced *E*/*Z*‐isomerizations [1, 2, 3]. Herein, we outline examples of *E*/*Z*‐isomerizations where supramolecular assemblies have a direct impact on the observed outcome of the photochemical reaction behavior in aqueous media. Although there are many examples of *E*/*Z*‐isomerizations in organic solvents [4, 5], the reaction behavior is often not transferable to biological systems and applications; therefore, photoswitchable molecules that can be efficiently controlled in water are of interest [6, 7].

However, challenges arise when using water as the solvent, such as limited solubility [8, 9], molecular aggregation [10, 11], or loss of photoswitching properties [12, 13]. Supramolecular interactions influence multiple aspects, including the switching efficiencies [14, 15, 16], water‐solubilities [17, 18], the selectivity of the isomerizations [12, 19], or stabilities and the number of reversible switching cycles [14, 20]. Hence, supramolecular systems represent a compelling strategy to influence the outcome of *E*/*Z*‐isomerizations through interactions and distinct local environments. This review aims to illustrate the importance of interactions between small molecules that undergo *E*/*Z*‐isomerization and supramolecular entities rather than being comprehensive. Comprehensive reviews focusing on *photoswitches in water* [21] or various *applications of photoswitches in supramolecular assemblies* [22] have been published elsewhere.

The importance of interactions and the local microenvironment, which is decisive for reaction behavior and outcome, is highlighted through studies on *E*/*Z*‐isomerizations in association with supramolecular entities such as macrocages [23, 24] or micelles [25]. The host–guest interaction is a special stereoelectronic rearrangement between the molecules through non‐covalent interactions [26] and depends on multiple factors such as size, shape, and conformation of the molecules, the presence of charges [27]. Based on the surrounding media, the hydrophilicity and hydrophobicity of the molecules are also effective [28]. Photoisomerization not only results in a change in photophysical properties of photoswitches but may also alter the geometry, polarity, or flexibility of the molecules [29].

The encapsulation of photoswitchable molecules provides a promising strategy for the controlled release of guests [30], also in aqueous media, as it is challenging to develop controllable host–guest systems by an external stimulus [31]. Using supramolecular structures in water as the medium to alter reaction outcomes can be perceived as a biomimetic strategy [32]. These artificial systems are easier and simpler to study than biological systems; however, effects of interactions with supramolecular structures and the microenvironment can be expected to play important roles when studying biological functions or the behavior of therapeutics [33]. Moreover, artificial photoswitchable systems allow for the elucidation of key processes in living organisms and pave the way for potential applications by mimicking biological systems [34, 35]. The importance of the selective association of a free molecule to an assembly with biological entities is illustrated in selected biological applications. *E*/*Z*‐isomerizations can be applied, for example, to control ON–OFF states [36] or to induce changes in properties or behavior which can affect biological functions [37, 38, 39]. The elegant and useful feature of a controlled reversible reaction is that, in addition to pH [40], redox‐sensitive [41], thermal [42], and solvent [43] or mechanically induced switches [44], one can also switch between states using light [45]. The use of light offers a high level of spatiotemporal control through narrow wavelength selection. Wavelengths in the range from visible to near‐infrared (near‐IR) offer deeper penetration and lower phototoxicity [46] compared with *UV*‐light, which is of high importance for therapeutics and studying biological functions [47]. The newly developing strategy of the utilization of *E*/*Z*‐photoswitches in photodynamic therapy (PDT) and photothermal therapy offers a promising approach for photocontrolled generation of reactive oxygen species [48, 49, 50] and enhanced photostability of photosensitizers [51]. In the following, representative examples that target the structure and function of RNA, DNA, histones, bacteria, enzymes, ion channels, neural systems, cell membranes, and drug delivery systems have been illustrated in order to highlight the importance and diversity of *E*/*Z*‐isomerizations in biomimetic and biological media.

## 2 Results and Discussion

### 2.1 *E*/*Z*‐Isomerization in Confined Environments

Photoswitches are molecules that can effectively undergo structural or conformational changes in response to light exposure at certain wavelengths. These transformations induce controlled and reversible modifications in distinguishable optical or chemical properties [52]. Photoswitches typically convert between two forms or sometimes even more, such as *E* ↔ *Z* [4], spiropyran ↔ merocyanine isoforms [53], or aromatization switches [54]. A supramolecular host is a molecule or molecular assembly that can bind a guest molecule *via* non‐covalent interactions, resulting in a host–guest complex [55]. The influence of the host on the guest extends beyond binding and can modify the physical, chemical, electronic, and biological properties of the guest [56, 57, 58].

#### 2.1.1 Macrocyclic or Capsule Confinement

Supramolecular confined environments enable the modulation of photoisomerization in a controllable manner in aqueous environments. Such confined environments can provide selectivity toward certain isomers, accelerate isomerization reactions, and enhance isomerization efficiency. Host–guest complexes offer a means to overcome the water solubility issues of substrates and the requirements of longer wavelengths for biological applications.

**Figure 1:**
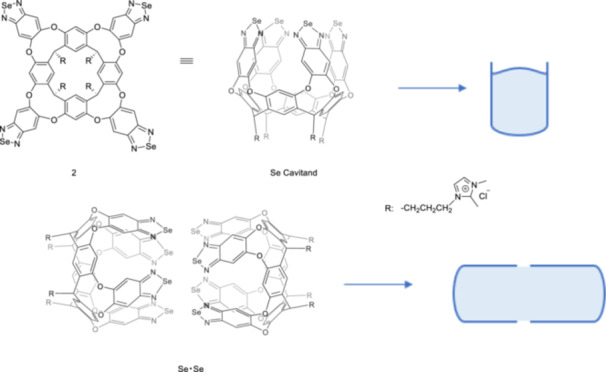
The structures of Se cavitand and structural representations of Se cavitand and Se · Se capsules formed through chalcogen bonds, which provide selective separation of *Z‐* **1** isomers by simple extraction.

A self‐assembled Se cavitand forms water‐soluble Se・Se capsules through chalcogen bonding whose structural representations are given in Figure 1 [20]. The way in which guests may interact with the capsules might resemble their interaction within biological receptors. These capsules exhibit high binding selectivity for *Z‐* **1** isomers of free alkyl‐*O*‐methyl aldoximes (R − C(H)=NOMe) with different chain lengths and functional groups. In comparison, the use of organic solvents leads to approximately 60% *E*‐configuration and 40% *Z*‐configuration for various free derivatives.

**Figure 2:**
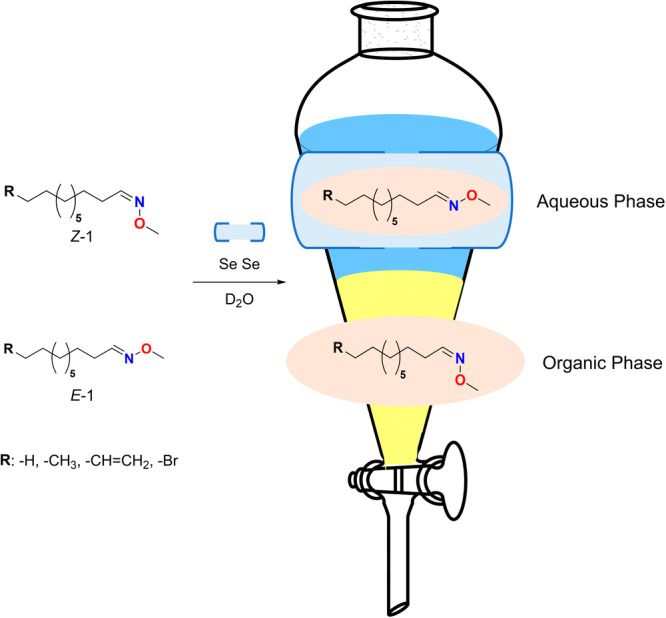
Representation of separation of *E*/*Z‐* **1** isomers through simple extraction by selective encapsulation of *Z‐* **1** within Se · Se capsules.

The separation of *E*‐**1** and *Z‐* **1** isomers can thus be achieved through simple extraction, where the aqueous layer contains the encapsulated *Z‐* **1**‐isomers, while the uncomplexed *E*‐**1** isomers remain in the organic layer (Figure 2). The high selectivity of confinement was attributed to conformational restrictions and the reduced stability of *E*‐**1** isomers within the hydrophobic cavity of the Se · Se capsules.

**Figure 3:**
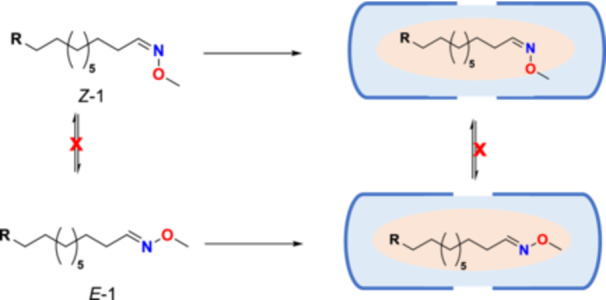
Se · Se capsule‐mediated isomerization of guest molecules is selective towards *Z*‐isomers.

The confined environment significantly affects the *E*/*Z*‐isomerization in favor of *Z‐* **1** isomers (Figure 3) at room temperature. The isomerization reactions were monitored by ^1^H‐NMR spectroscopy in D_2_O. Sonication accelerated the rate of isomerization reactions by about 8–10‐fold. Notably, the reusability of Se cavitand **2** demonstrated no decrease in selectivity toward *Z‐* **1** isomers after more than six recovery cycles.

**Figure 4:**
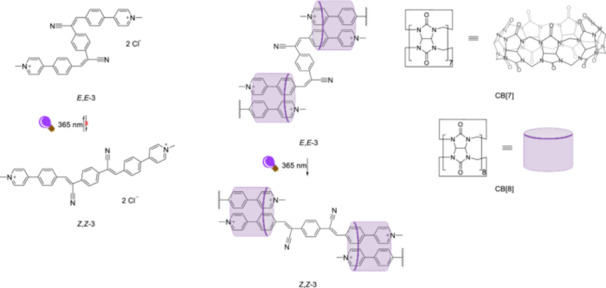
Once *E,E‐* **3** is formed upon irradiation of *Z,Z‐* **3** at 365 nm, it cannot revert to *Z,Z‐* **3** in the free state, and in CB[8] *E,E‐* **3** can be converted to *Z,Z‐* **3** upon irradiation at the same wavelength. CB[8] represented as a purple cylindrical cartoon shape.

Cucurbit[n]urils are one of the most commonly used macrocyclic host molecules in supramolecular chemistry, thanks to its applicability in aqueous environments [59]. Controling self‐assembled morphologies by light is of interest in designing smart materials, as investigated in a recent study by Zhang and Liu et al. [60]. They explore the *E*/*Z*‐isomerization of a cyanostilbene derivative (Figure 4) in a host–guest complex formed with cucurbit[8]uril (CB[8]) in aqueous solution. The photoisomerization of the *Z,Z‐* **3** of a cyanostilbene derivative isomerizes irreversibly when irradiated with light at 365 nm. The isomerization reaction was monitored using absorption and fluorescence spectroscopy in water, as well as ^1^H‐NMR spectroscopy in D_2_O and high‐resolution mass spectrometry (HRMS) to rule out the possibility of cyclization or dimerization reactions. Although the *Z*,*Z*‐configuration irreversibly isomerizes to the *E*,*E*‐configuration in the free state, the *E,E‐* **3** can return to the *Z,Z‐* **3** after it forms a host‐complex with CB[8] under irradiation at the same wavelength. However, CB[7] with a smaller cavity could not facilitate the photoisomerization of the *E,E‐* **3** back to the *Z,Z‐* **3**. The transmission electron microscopy (TEM) image of the free *Z,Z‐* **3** displayed a filamentous shape. After the sample was irradiated, its shape changed to that of small fragments, indicating that the *E*,*E*‐configuration has a different morphology. When the *E,E‐* **3** is incorporated into the CB[8] macrocycle, further irradiation of the sample causes it to revert to a filamentous shape, exhibiting the same topography as the host‐guest complex of the *Z,Z‐* **3** with CB[8].

**Figure 5:**
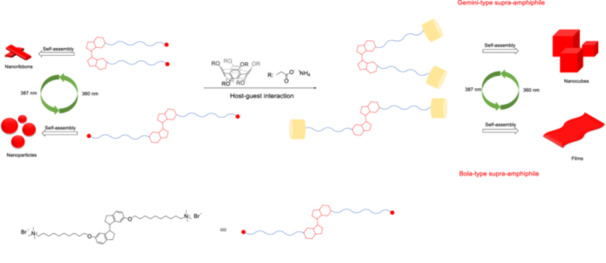
The stiff‐stilbene‐based amphiphilic guest undergoes morphological change from nanoribbons to nanoparticles while switching between *Z*‐isomer and *E*‐isomer and gemini‐type to bola‐type transformation upon host–guest interaction with pillar[5]arene under irradiation.

Another example for light‐triggered changes in morphology is the combination of pillararenes and stiff‐stilbenes to provide a photocontrolled gemini‐type to bola‐type transformation (Figure 5) for a supra‐amphiphile [61]. A water‐soluble pillar[5]arene and a stiff‐stilbene derivative exhibit photo‐responsive assembly behavior based on host–guest interactions and light. The stiff‐stilbene‐based amphiphilic guest undergoes *Z* **→** *E* isomerization, resulting in the morphological change from nanoribbons to nanoparticles upon irradiation by *UV*‐light at 387 nm in water. The *Z*‐isomer self‐assembles into nanocubes in the presence of pillar[5]arenes. The irradiation of the host–guest complexes of *Z*‐isomers at the same wavelength induces the conversion of the gemini‐type supra‐amphiphile into the bola‐type and morphological change to crumpled films.

**Figure 6:**
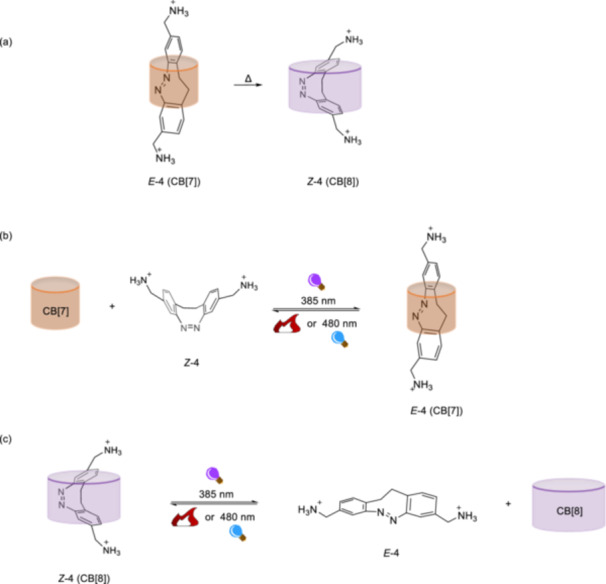
(a) Thermal isomerization *E* → *Z* is significantly accelerated by the presence of CB[7] or CB[8] types of cucurbiturils. (b) Light‐induced interconversion between *Z*‐ and *E*‐isomers in the presence of CB[7], showing preferential binding of CB[7] to the *E*‐**4** isomer. (c) The reversible binding of *Z*‐**4** in the CB[8] complex, with a 1:1 binding ratio, highlights the stronger affinity of CB[8] for the *Z*‐**4** isomer compared with the *E*‐**4** isomer.

CB[n] (e.g., CB[5], CB[6], CB[7], and CB[8]) can function as hosts for diazocine guest molecules to achieve photoswitchable host–guest complex systems in aqueous solution as illustrated by for example Pischel, Herges and Basílio et al. [62] Diazocines are intriguing bridged azobenzene derivatives formed by connecting two phenyl rings through an ethylene chain [63]. The resulting eight‐membered core structure leads to the *Z*‐**4** being thermodynamically stable, contrary to the parent azobenzene. The water‐soluble bis‐aminomethyl‐substituted diazocine derivative developed in the study [62] fits well into the cavity of CB[7] in its *E*‐isomeric form. However, due to its larger cavity, CB[8] binds more readily to *Z*‐**4**, demonstrating higher affinity and much stronger selectivity for it compared with CB[7]. ^1^H‐NMR studies indicate that the aromatic and the bridge methylene protons of the *E*‐**4** shift significantly upfield. This shift was attributed to the central diazocine moiety being deeply hosted in the cavity of the CB[7] macrocycle. However, the slight downfield shift of ^+^H_3_NCH_2_ protons suggests that this substituent is oriented outside the cavity, as shown in Figure 6. The interaction of *E*‐**4** with the CB[7] macrocycle also resulted in a red shift in the absorption maximum of the *E*‐isomer. In the case of the *Z*‐isomer, all protons are shifted upfield upon interaction with the CB[8] macrocycle. These changes in the ^1^H‐NMR signals were attributed to an optimal fit of the *Z*‐**4** into the macrocycle cavity as illustrated in Figure 6. Photoisomerization from the *Z*‐**4** to *E*‐**4** upon irradiation with 385 nm light leads to a similar photostationary state (PSS) distribution, regardless of the presence of any macrocycles. The efficiency of *E* → *Z* photoisomerization in water, facilitated by light above a wavelength of 480 nm, remains unaffected by the formation of host–guest complexes.

However, CB[7] and CB[8] macrocycles individually accelerated the thermal *E* → *Z* isomerization without product inhibition (Figure 6a). No significant change in the rate of thermal isomerization was observed in the presence of CB[5] and CB[6] compared with unbound *E*‐**4** in water, or when a stronger binding guest for CB[7] was included as control experiments. This indicates that the observed acceleration is due to the formation of the host‐guest complex.

**Figure 7:**
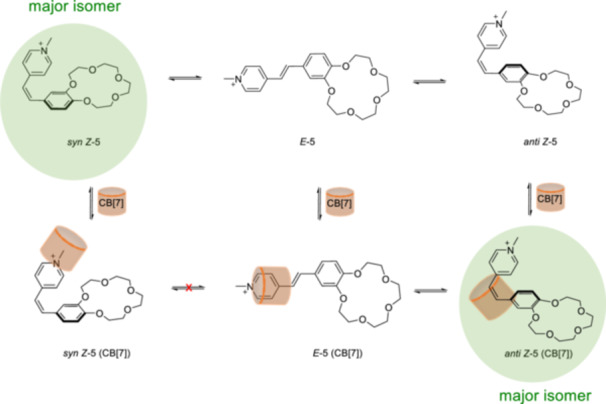
The photoisomerization of *E*‐**5** in the absence and presence of CB[7]. The favored products are shown in circles.

Supramolecular stereorecognition by the achiral CB[7] host in water was achieved with a styrylpyridinium dye bearing an benzo‐15‐crown‐5 ether that has an *anti* and *syn* conformer [64]. According to NMR spectroscopy studies, when the thermodynamically stable *E*‐**5** is irradiated by light at a wavelength above 300 nm, the ratio of *E*/*Z* isomers is 31:69, with a 78:22 ratio for the *syn* and *anti‐*conformers of the *Z*‐**5** in the free state. The presence of CB[7] leads to a shift in stereoselectivity toward the *anti Z*‐**5** (Figure 7), as well as enhancing the *E* → *Z* conversion. Although slow photobleaching of the dye was observed when irradiated with 313–405 nm alternating light cycles in the free state in aqueous solution, no photobleaching of the molecule was detected in the presence of CB[7]. According to the competitive studies with Ba^2+^, conformational memory is retained. The *anti* Z‐**5** conformation remains after the dissociation of the host–guest complexation.

**Figure 8:**
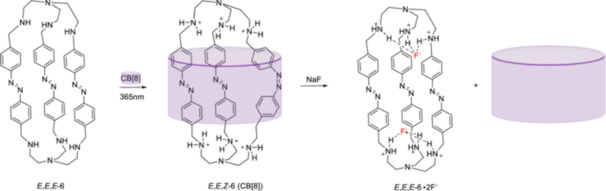
Photoisomerization of *E*,*E*,*E*‐**6** to *E*,*E*,*Z*‐**6** with high selectivity enabled by CB[8] in a pH 3.6 buffer.

Controlling the photoisomerization of multiple switching units in one molecule is of great interest since it is mimicking the role of chaperones, natural enzymes that assist the correct folding of large proteins [65]. Anion binding is able to control the encapsulation of azoazacryptand bearing three switching azobenzenes in CB[8] in an aqueous environment [12]. The *E*,*E*,*E*‐**6** of cryptand readily converts to *Z*,*Z*,*Z*‐**6** upon irradiation in organic solvents at 365 nm. However, in aqueous media, a complex distribution of different isomers was observed, and all‐*E*‐**6** not only undergoes photoisomerization to all‐*Z*‐**6**. Host–guest complexation with CB[8] as well as fluoride and perchlorate ions, were shown to affect isomer distribution. The irradiation of *E*,*E*,*E*‐**6** in the presence of CB[8] resulted in the formation of CB[8] encapsulated *E*,*E*,*Z*‐**6** as the major product (70%, Figure 8), and 20% of the *E*,*Z*,*Z*‐**6** isomer inside CB[8] in a pH 3.6 acetate buffer. The interaction between guest molecule *E*,*E*,*Z*‐**6** and the CB[8] host primarily depends on the cation–dipole interactions and hydrogen bonding between carbonyl groups of the host and protonated amine units of the guest. Upon the addition of fluoride ions, the guest molecule is released by disrupting the hydrogen bonding between the host and guest.

*Z*‐Cinnamic acid was found to promote the activity of plant growth, whereas the *E*‐form is inactive [66]. Therefore, it is of interest to control and tune the photoisomerization of cinnamic acid and its methyl ester by host–guest complexation in aqueous solution. Complexation with CB[7] enhanced the efficiency of the *E* → *Z* photoisomerization of cinnamic acid and the stability of the product [67].

Photoswitches that have different fluorescent properties according to the isomers can be used as fluorescent indicators in microscopic imaging. Therefore, it is interesting to study the effect of complexation in water‐based systems. Kubinyi et al. [68] used a cationic stilbene‐type photoswitch in confined environments formed by the anionic macrocycles carboxylato‐pillar[5]arene and carboxylato‐pillar[6]arene. Complexation with carboxylato‐pillar[6]arene enhances the quantum yields for photoisomerization processes in both directions of the stilbene‐type molecular photoswitch, as well as increases the fluorescent quantum yield of the *E*‐Isomer.

**Figure 9:**
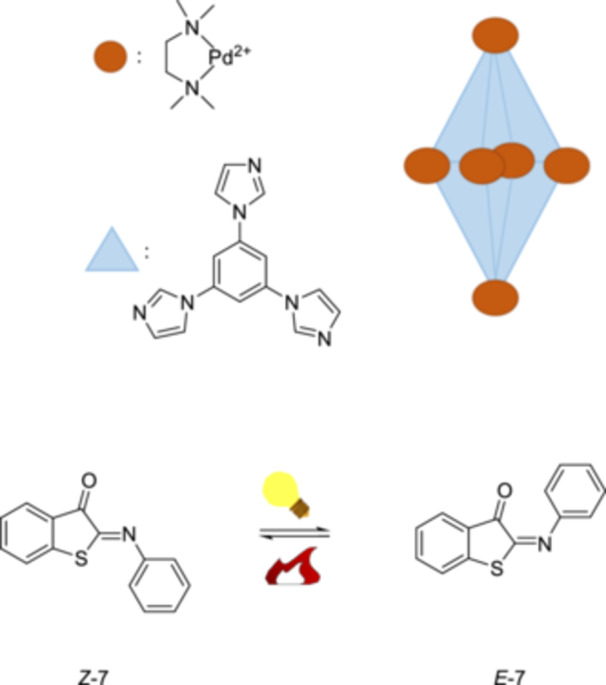
The metal‐organic cage and the photoisomerization of *Z*‐**7** to *E*‐**7** and thermal back‐isomerization *E*‐**7** to *Z*‐**7**.

A Pd‐based metal‐organic cage enhances the water solubility of an iminothioindoxyl **7** photoswitch, while enabling photoreactivity in water (Figure 9) [17]. Thereby, the ultra‐fast *E*/*Z*‐isomerization property is retained, and the thermal relaxation of the *E*‐isomer (3 ms) occurs significantly faster than in the free state in MeOH (18.5 ms). It has been demonstrated that the confined environment modulates the excited state dynamics in water, in contrast to the dynamics that occur in organic solvents. The excited state bifurcates to distinct pathways, indicating that the excited state dynamics and molecular switching behavior can be controlled by changing the supramolecular environment through confinement.

Biologically active amines can be detected with the pH‐sensitive azobenzene dye complexation with CB[7] [69]. The CB[7]‐mediated *E*/*Z*‐isomerization produces an intense purple color in certain azobenzene dyes that exhibit high association constants (Ka) with the hosts. These properties were employed in indicator displacement assays to differentiate biologically active amines, including ephedrine, pseudoephedrine, l‐adrenaline, nicotine, dopamine, caffeine, and acetylcholine in water. Among CDs, including α‐cyclodextrin (α‐CD), β‐CD, and γ‐CD, γ‐CD improves the water solubility of azobenzene‐based probes more effectively and has a smaller impact on the pH probe performance of azobenzenes. This is because γ‐CD has a larger cavity, leading to weaker host–guest interactions that minimize interference with pH probing [70].

The controlled dimerization of β‐CD equipped with azobenzene into the secondary face was studied [71]. Water‐soluble triazoylazobenzene motif of the developed azobenzene forms an inclusion complex within β‐CD, whereas when the azobenzene is covalently attached to the CD, the inclusion is prevented. The photocontrolled *E*/*Z*‐isomerization of *E*‐isomer content higher than 95% partially proceeds upon 365 nm irradiation, and the PSS state of 1:4 was determined by ^1^H‐NMR. Back isomerization from a *Z*‐isomer to an *E*‐isomer can be achieved thermally over a longer period of time. A β‐CD with an azobenzene‐derivative covalently attached in the *E*‐configuration enables dimerization in an aqueous environment. The dimerization is stabilized by interactions between aromatic rings as well as partial reciprocal inclusion into the β‐CD cavity. Photoisomerization to *Z*‐isomer converts dimers into monomeric forms, offering a promising spatiotemporal control strategy for self‐assembly. Notably, induced circular dichroism studies reveal that in the presence of the *E*‐isomer, certain derivatives display a bisignal, indicating exciton coupling and suggesting proximity between the azobenzenes. Pseudopolyrotaxane‐type supramolecular architectures form through the complexation of bis‐azobenzene with γ‐CD at elevated γ‐CD concentrations. *E*/*Z*‐photoisomerization also disrupts these supramolecular interactions. These light‐responsive self‐assembly systems have potential applications in nucleic acid delivery systems. The self‐assembling system consists of a water‐soluble bis‐azobenzene derivative or its monotopic analogous and α‐CD, β‐CD, or γ‐CD were investigated as hosts [72]. *E*‐isomers of both bis‐ and mono‐azobenzene derivatives demonstrate high affinity for α‐CD, while *Z*‐isomers exhibit significantly lower affinity. Consistent with previous findings, complexation of the *E*‐isomer of the bis‐azobenzene derivative with α‐CD induces dimerization, and self‐assembly of these complexes is disrupted following *E*/*Z*‐photoisomerization. In contrast, both *E*‐ and *Z*‐isomers can be encapsulated by β‐CD or γ‐CD, although there is only moderate selectivity for the *E*‐isomer.

To address the problem of *UV*‐light excitation of azo‐based systems [73, 74], a supramolecular strategy known as disequilibration by sensitization under confinement (DESC) was developed [75, 76]. In a recent implementation of DESC, the assembly of arylazopyrazole‐incorporated surfactants and photosensitizers in a confined environment is used to modulate surface tension at air–water interfaces. Heterodimer formation of surfactants and BODIPY derivatives provides reversible isomerization under irradiation varying between green and blue light. A phenoxazine‐based dye also enables reversible photoswitching for surfactants between low and high‐surface‐tension states, alternating between red and green light. The authors further proposed that the strategy could be applicable to other platforms, such as hydrogels and solid–water interfaces, as well as in biological applications [76]. In a particular application of DESC, the strategy integrates a macrocyclic host with a photosensitizer to selectively bind and sensitize *E*‐azobenzenes, facilitating their isomerization. Herein, DESC relies on the encapsulation of the *E*‐isomer of azobenzene, undergoes isomerization, and a photosensitizer that utilizes visible light energy to drive the *E*/*Z*‐isomerization. After the photosensitizer absorbs light, promoting it to the excited state, it undergoes intersystem crossing to the triplet state with enhanced efficiency under confinement. Triplet energy transfer from the photosensitizer to the azobenzene is favorable due to the dynamics of the azobenzene (i.e., the process is largely entropy driven) [77]. The non‐planar structure of the *Z*‐isomer enables the azobenzene release from the complex as the host system is selective toward the *E*‐isomers.

#### 2.1.2 Supramolecular Aggregates in Aqueous Environments

Supramolecular aggregates are assemblies of molecules formed through noncovalent intermolecular interactions such as hydrogen bonding, π–π stacking, van der Waals forces, or electrostatic interactions. These aggregates are formed reversibly and spontaneously, and are often responsive to external stimuli, such as light [78, 79, 80].

**Figure 10:**
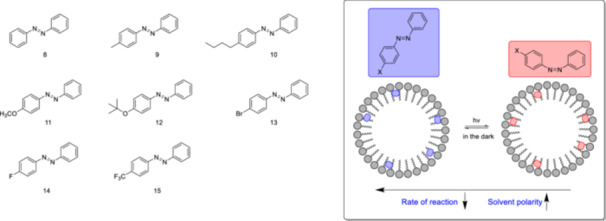
The chemical structures of azobenzene and its derivatives, whose photoisomerization kinetics have been investigated in organic solvents and micellar aqueous environments, and the representation of *E*/*Z* isomerization in an aqueous micellar environment. Potential model reactions for probing the polarity of the water‐surfactant interfaces.

In micellar environments, *E/Z* isomerization of azobenzene and its derivatives is a promising model reaction to probe the polarity of the water‐surfactant interfaces. Therefore, the kinetics of photoisomerization in polar organic and micellar media, specifically the *E*/*Z*‐isomerization, were investigated by Maria et al. [81] in 2009. The study focused on the photoisomerization of parent azobenzene, and its 4‐substituted derivatives **8–15** (Figure 10) in polar organic solvents, their mixtures with water, and aqueous micelle solutions at room temperature. Azobenzene and its analogs are thermodynamically more stable in the *E* configuration and can undergo isomerization of *E* → *Z* upon irradiation by *UV*‐light. The back‐isomerization from *Z* to *E* toward the more favorable configuration (*E)* could be driven by visible light or thermally [82, 83]. The low solubility of the investigated azobenzene derivatives (**8–15**) in water is addressed through interactions with octaethylene glycol monododecyl ether (C_12_E_8_), sodium dodecylsulfate (SDS), and dodecyl trimethylammonium bromide (C_12_TAB) micelles [81]. The guest molecules are thought to be located in the interfacial region of the micelles, where the reaction could occur due to their reduced mobility. The rate of *Z* to *E* isomerization is inversely proportional to solvent polarity, with the exception of the *n*‐butyl derivative **10**, which was associated with higher sensitivity of the *n*‐butyl group toward solvation and hydrophobic effects. When considering the *Z* to *E* isomerization reaction in micellar solutions, the reaction rate is lower in charged micelles formed by negatively charged SDS and positively charged C_12_TAB compared with neutral micelles created with C_12_E_8_ (exception the *n*‐butyl derivative **10**). The change in V‐shaped Hammett plots for the derivatives **8–15** (Figure 10) in MeOH and EtOH to a linear Hammett plot in water demonstrates that the inversion pathway is suppressed in water.

The Hammett plots obtained from molecules **8–15** yield different *ρ* values in various ionic and non‐ionic micellar environments. For example, in the presence of ionic micelles like CTAB and SDS, the *ρ* values are approximately −2.4 and −2.1, respectively. These values are more negative compared with the *ρ* value obtained in a homogeneous EtOH solution, which is around −1.6. The difference between EtOH solution and non‐ionic micellar environment (e.g., *ρ*: ≈ −1.8) is not drastically different from an ionic micellar environment.

**Figure 11:**
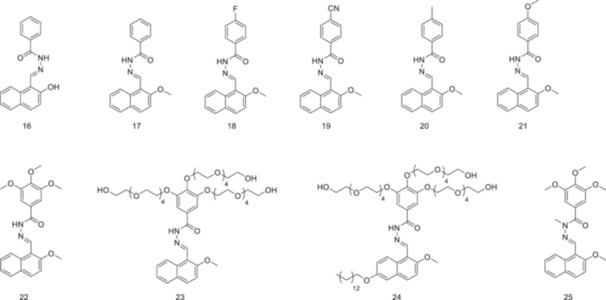
The structures of benzoylhydrazones, whose photoswitchable properties have been investigated.

*Z*‐isomers of hydrazones do not have sufficient stability and are generally stabilized through an intramolecular hydrogen bond [84]. Yuan and Zheng [85] developed a hydrazone‐based photoswitchable system that shows high *E* → *Z* conversion efficiency without the requirement of an intramolecular hydrogen bond. The observed photoswitching efficiency was high, whereas hydrazone‐based systems often experience low conversion efficiencies, from the *E*‐isomer to the *Z*‐isomer [84]. Apart from hydrazone derivative **16**, all other *E*‐hydrazones **17–25** (Figure 11) undergo *E* → *Z* photoisomerization upon irradiation at 365 nm in organic solvents.

The derivative **23** (Figure 11) retains its photoswitching property in water, and dynamic light scattering and cryo‐TEM studies demonstrate that it forms nanoparticles in water. The hydrazone derivative **24** (Figure 11) shows similar photophysical properties, and *E*‐**24** can be converted to *Z*‐**24** upon irradiation upon 365 nm irradiation, and it also forms nanospheres in water. Both molecules have the potential to serve as hosts for a photocontrollable drug delivery system. To that end, the controlled release of the anticancer drug procarbazine (PCZ) was tested in the presence of nanoparticles of **24**. The water solubility of PCZ is very poor, approximately 0.002 mg/mL. However, its solubility increases to 0.45 mg/mL in the presence of **23**. When the drug‐loaded nanoparticles were irradiated at 365 nm, new aggregates with a diameter ranging from 1.10 to 1.60 μm were observed *via* scanning electron microscopy.

**Figure 12:**
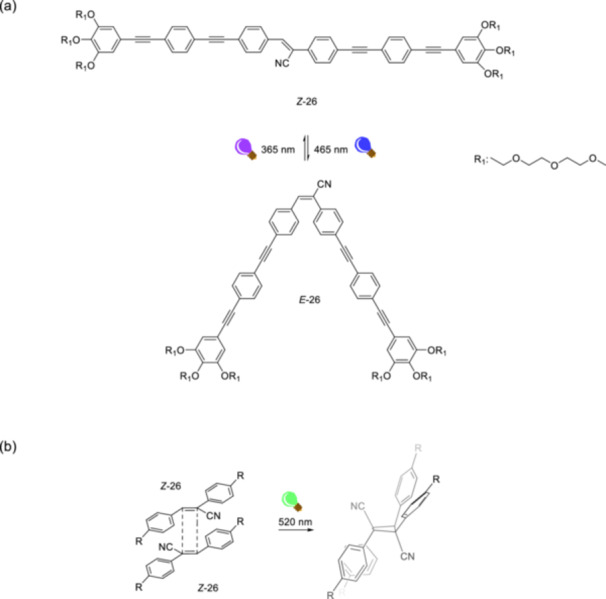
(a) Photoisomerization of cyanostilbene bolaamphiphile (**26**) upon irradiation alternating between 465 and 365 nm in organic solvents. (b) Schematic representation of [2 + 2] photocycloaddition reaction between *Z*‐**26** isomers into a single cyclobutane unit (*anti* head‐to‐tail) by supramolecular polymerization in an aqueous environment upon irradiation with visible light.

To obtain a tunable solvent‐dependent photochemical system that overcomes the complexity of competing photochemical and supramolecular pathways, Fernández et al. [86] designed a cyanostilbene bolaamphiphile. In organic solvents, the molecule undergoes reversible *E*/*Z*‐isomerization (Figure 12a), whereas in an aqueous environment, a near‐quantitative [2 + 2] photocycloaddition reaction (Figure 12b) *via* supramolecular polymerization preferentially occurs. The polymerization reaction in aqueous media was assigned to a preorganized antiparallel J‐type arrangement of the cyanostilbenes in an aqueous environment, whereas a monomeric state in bulk organic solvents was proposed.

When the photoisomerization reaction is monitored using *UV*‐vis absorption spectroscopy at incremental irradiation times, an isosbestic point is observed, indicating that only two isomers are present (Figure 12a). Irradiation at 465 nm promotes the isomerization toward *E*‐**26** in the photoequilibrium state with *Z*:*E* ratio of 21:79 in MeOH. *UV* irradiation at 365 induces *E* → *Z* back‐isomerization reaching *Z*:*E* ratio of 66:34 in MeOH. Alternating irradiation between 465 and 365 nm shows no degradation, even after 20 consecutive cycles. In an aqueous environment, it is presumed that the cyanostilbene molecules are present in an aggregated state that allows for close interactions, paving the way for optimal proximity to trigger the photocycloaddition reaction. *Z*‐**26** has a greater tendency than *E*‐**26** to form J‐type aggregates that undergo the cycloaddition reaction with optimal geometry since J‐type exciton coupling is more pronounced for the *Z*‐**26** isomer. The measured emission lifetimes (e.g., 1.674 ns in H_2_O for *E*‐**26** and 4.471 ns in H_2_O for *Z*‐**26**) and hypochromism as the spectral change in water for *E*‐**26** isomers indicate a less ordered structure in aqueous solution. The cycloaddition reaction products were characterized by Gel Permeation Chromatography and HRMS studies, confirming cyclobutane ring formation. High‐performance liquid chromatography with a chiral stationary phase revealed that only *the anti* head‐to‐tail regioisomeric form of cyclobutane is formed as a result of the cycloaddition reaction.

**Figure 13:**
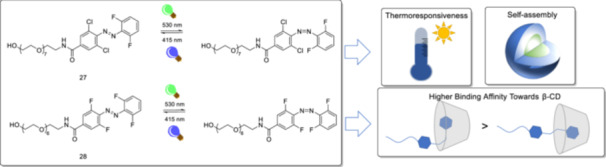
The reversible photoisomerization of water‐soluble tetra‐*ortho*‐substituted azobenzene derivatives under visible light, and self‐assembly behavior and complexation with β‐CD. *Z*‐isomers show high thermal stability.

The photoisomerization of tetra‐*ortho*‐substituted azobenzene derivatives that can reversibly self‐assemble was studied in water using visible light [87]. This study represents an unprecedented example of all visible light photoswitchable system that shows reversible complexation with β‐CD and self‐assembly behavior (Figure 13). The *Z*‐isomers of both molecules exhibit long thermal lives (i.e., more than 300 days) at room temperature. Irradiation of dark‐adapted compounds of **27** and **28** at 530 nm for 15 min (30 mW·cm^2^^−^) leads to PSS values for *Z*‐isomers 67% and 72%, respectively. PSS values for **27** and **28** *E*‐isomers are 84% and 67% after irradiation at 530 nm for 5 min (90 mW·cm^2^^−^). Solutions of **28** above the critical micelle concentration (CMC) exhibited the lower critical solution temperature. There is a 10°C difference between cloud temperatures for *Z* and *E*‐isomers of **28**, enabling photocontrolled aggregation under visible light. The reversible complexation between azobenzene derivatives and β‐CD was also observed, and *Z*‐**28** exhibited a higher binding constant with β‐CD than the *E*‐isomer (Figure 13). For **27** derivatives, only modest differences in binding constants between *Z* and *E*‐isomers were detected.

Azo‐based photoswitches can suffer from reduced stability in cellular environments due to the presence of compounds like glutathione. Glutathione is present in cells at a concentration range of 0.5–10 mM and can reduce azo‐based photoswitches, depending on their structure and redox potentials, thereby inactivating them [88, 89]. The effect of substitution on *E*/*Z*‐isomerization and the stability against glutathione of water‐soluble arylazopyrazoles, along with a light‐responsive host–guest interaction of the arylazopyrazoles with β‐CD, have been investigated [90]. The substitution enables fine‐tuning of photoswitching performance and binding to β‐CD, while preserving the stability of arylazopyrazoles against a concentrated solution of glutathione (i.e., 10 mM, the highest cellular concentration), making these systems particularly promising for biologically relevant and in vivo applications. Another study by the same group [91] demonstrates that introducing a carboxylic acid group into arylazopyrazole enhances water solubility and enables structural modifications for subsequent derivatization. Water‐soluble arylazopyrazole derivatives exhibit improved switching properties, increased thermal stability of *Z*‐isomers, and form photoresponsive complexes with β‐CD. As such, the limitations of azo‐based photoswitches were addressed including incomplete switching [92], and low thermodynamic stability of *Z*‐isomers [93].

Photo‐responsive micelles can be achieved by the incorporation of arylazopyrazoles into block copolymers [94]. These micelles with an appropriate derivative can undergo a morphological change upon *E*/*Z*‐isomerization, transforming from spherical to cylindrical micelles in water when irradiated with green (520 nm) and *UV*‐light (365 nm), whereas most derivatives form spherical micelles in both isomeric states.

Supramolecular self‐assembly behavior can play a critical role in effective *E*/*Z*‐photoisomerization. Supramolecular interactions strongly influence the bio‐applicability of photo‐responsive systems and govern optically controlled morphological changes.

### 2.2 Biological Applications of *E*/*Z*‐Isomerizations

#### 2.2.1 Monitoring Biological Systems Through Fluorescence

Fluorescence‐based approaches for monitoring biological systems provide strategies with high sensitivity, selectivity, and spatial resolution [95, 96]. The incorporation of photoswitchable molecules into fluorescent systems offers an additional significant advantage, namely, spatiotemporal regulation of supramolecular interactions and signaling events.

**Figure 14:**
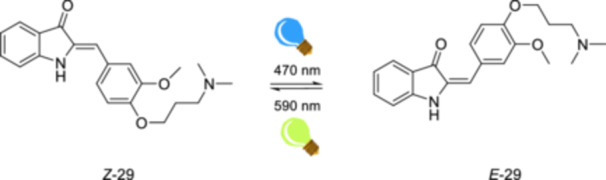
Reversible photoisomerization of hemi‐indigo derivative **29** upon visible light irradiation.

A study by Berdnikova [97] demonstrates that hemi‐indigo derivatives can serve as a novel light‐controllable RNA protection platform. Hemi‐indigo derivative **29** undergoes *E*/*Z*‐photoisomerization upon visible light irradiation alternating between 470 and 590 nm in water (Figure 14). The fluorescence of *Z*‐**29** increases significantly when bound to the two elements of human immunodeficiency virus type 1 (HIV‐1) RNA (i.e., the transactivation response element (TAR) and the stem IIB region of the Rev response element (RRE‐IIB)) while both unbound isomers *Z*‐**29** and *E*‐**29** are non‐fluorescent in aqueous media. The increase in fluorescence of *Z*‐**29** upon binding was attributed to the restriction in rotational relaxation. After *Z*‐**29** binds to TAR or RRE‐IIB *Z*‐**29** isomerizes to *E*‐**29** upon irradiation at 470 nm and results in a significant decrease in fluorescence. Therefore, the system offers an ON‐OFF fluorescent system for RNA. According to Berdnikova [97], hemi‐indigo derivatives with photoswitchable characteristics in *E*/*Z*‐isomerization and fluorescence emission are promising candidates for monitoring biological systems.

The aggregation‐induced emission property of azobenzenes is a well‐established phenomenon with diverse applications [98, 99]. Fluorescent supramolecular aggregates can form through interactions between two non‐fluorescent components: adenosine 5’‐triphosphate (ATP) and an azobenzene‐guanidinium compound [100]. The water‐soluble azobenzene‐guanidinium exhibits strong affinity for nucleotides. Upon interaction with ATP, spontaneous assembly occurs in aqueous environments, including complex media containing DNA and proteins. These aggregates absorb light in the 320–460 nm range and emit photoluminescence at 650 nm. The photoswitchable nature of azobenzene‐guanidinium allows for light‐controlled assembly and disassembly of ATP aggregates using blue or *UV*‐light. Additionally, the formation of supramolecular aggregates can be regulated by introducing divalent metal ions or through enzymatic processes. The formation of ATP‐metal complexes and the enzymatic hydrolysis of ATP to adenosine and monophosphate by alkaline phosphatase both induce the disassembly of ATP‐azobenzene aggregates.

Fluorescent systems hold promise for monitoring biological systems with high sensitivity and resolution. The incorporation of photoswitchable molecules into such systems enables controlled monitoring. Photoswitchable fluorescent systems enable the observation of biological systems in a reversible and controllable manner with high sensitivity and spatiotemporal precision [101].

#### 2.2.2 Photomodulation of Gene Regulation and Cellular Signaling

**Figure 15:**
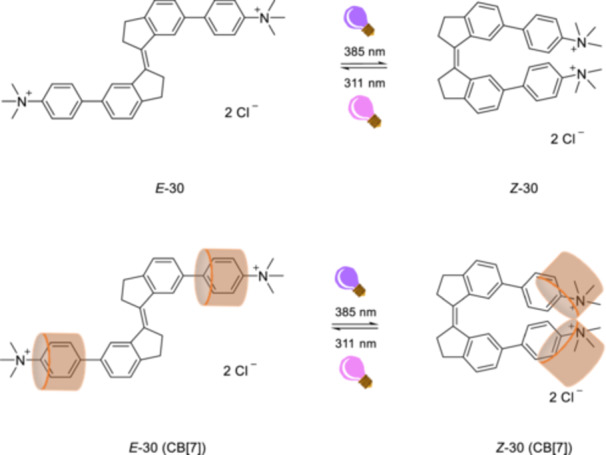
The reversible *E*/*Z*‐photoisomerization of water‐soluble stiff‐stilbene **30** and its host‐guest complex with CB[7].

Light‐controlled molecular tools provide platforms for investigating and modulating gene regulation and cellular signaling with high spatiotemporal precision. In biological systems, photoswitchable molecules reversibly control biomolecular interactions, protein function [102, 103], and gene expression pathways [104]. Yang, Yuan, and Liu et al. [105] investigated the photoisomerization of a water‐soluble stiff‐stilbene and its host–guest complex with CB[7], along with the fluorescence photoswitching characteristics. The *E*/*Z* isomers of stiff‐stilbene **30** can be reversibly switched by alternating irradiation between wavelengths of 311 and 385 nm. The host–guest complex of stiff‐stilbene 5 with CB[7] has still photoswitchable property at the same wavelengths. Job plot studies show that the stoichiometry of host–guest complexation is 2:1. The quantum yield for photoisomerization from *E*‐**30** to *Z*‐**30** is approximately 0.12 in the free state, but it decreases to around 0.06 upon complexation. Conversely, the quantum yield for *Z* → *E* photoisomerization is improved to about 0.07, increasing from approximately 0.03. The host–guest complex of stiff‐stilbene **30** and CB[7] (Figure 15) also exhibits photocontrollable fluorescence switching. The emission of the complex of *E*‐**30** with CB[7] at 440 nm irradiation was significantly quenched up to 70% upon irradiation at 311 nm for 120 s. Subsequent irradiation at 385 nm of the sample retained 59% of the initial emission intensity.

**Figure 16:**
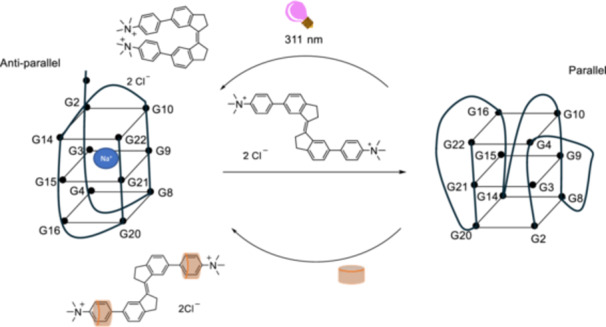
Schematic representation of the *E*‐**30** induced conversion of the G‐quadruplex parallel structure of Tel22 to an anti‐parallel structure.

G‐quadruplexes are involved in various biological processes such as transcription and replication. In particular, G‐quadruplexes could enhance or suppress transcriptional activity depending on their structures, which are parallel, anti‐parallel, or hybrid [106, 107]. The *E*‐**30** complex with CB[7] can regulate the human telomeric repeat, Tel22, which has an anti‐parallel structure in an aqueous solution. After incubating Tel22 with this complex, a structural change from anti‐parallel to parallel was observed (Figure 16), as confirmed by circular dichroism studies.

**Figure 17:**
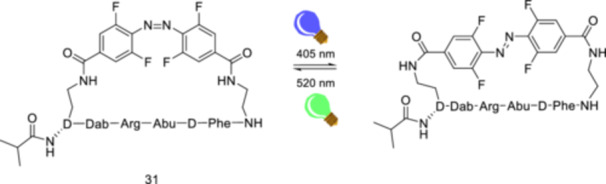
Photoisomerization of CS‐VIPS‐**31**, a WDR5‐targeted photoswitch that showed the most promising results among the CS‐VIP series.

Vázquez et al. [108] developed conformationally strained visible light photoswitches (CS‐VIPs), in particular CS‐VIPS‐**31**, that enable the regulation of hematopoiesis in vivo. CS‐VIPS‐**31** offers a bistable functional system that modulates the activity of the histone methyltransferase MLL1. MLL1 is an epigenetic regulator that activates gene expression through H3K4 histone methylation and controls gene expression in hematopoietic stem cells [109]. CS‐VIPS‐**31** enables the investigation of the hematopoietic function of the MLL1 complex. MLL1 leaves MLL1 − WDR5 complexes upon CS‐VIPS‐**31** binding to WDR5. The structures of WDR5 co‐crystallized with CS‐VIP‐**31** are promising models for studying azopeptides in an *E*/*Z*‐isomerization (Figure 17) in the crystalline state and their subsequent dissociation and reassembly from the protein target using time‐efficient techniques such as serial synchrotron crystallography. Specifically, *Z*‐CS‐VIPS‐**31** enables the inhibition of MLL1 activity in vivo, highlighting a possible toxicity mechanism of leukemia‐related cells depending on MLL1.

4,4′‐Diacetamido azobenzenes bearing amino substituents in the 2,2′‐positions were synthesized, showing that longer switching wavelengths with enhanced water solubilities upon substitution render azobenzenes potential photoswitches for biological applications [110].

**Figure 18:**
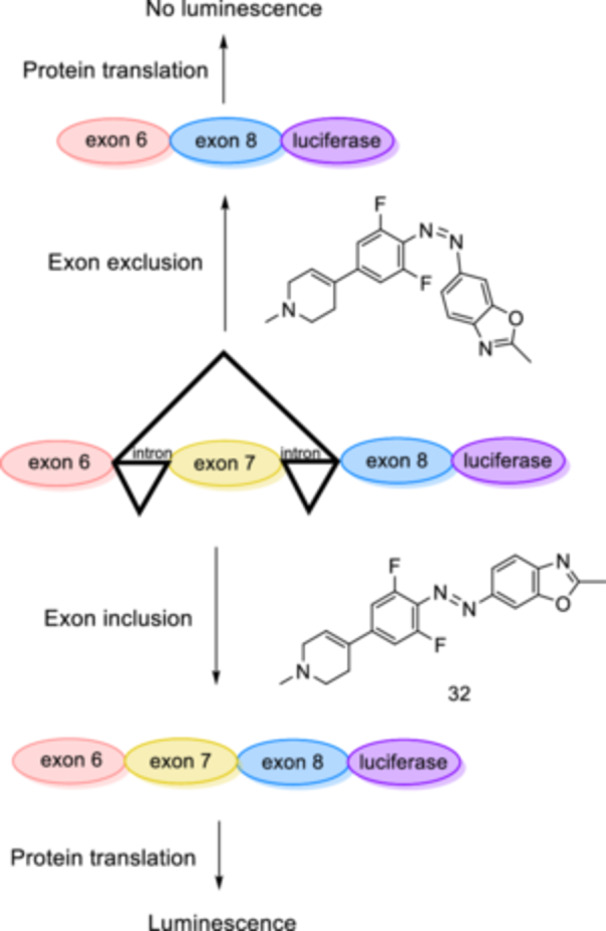
The *E* isomer of **32** promotes exon 7 inclusion when irradiated with green light, resulting in translation of the luciferase gene and luminescence emission. In contrast, the presence of the *Z*‐isomer of **32** causes the skipping of exon 7, abolishing luciferase translation and resulting in no luminescence.

Vázquez et al. [111] designed an azobenzene‐derived small molecule that enables the regulation of RNA splicing reversibly by light within primary patient‐derived cells. RNA splicing is an enzymatic process that plays a crucial role in proteome diversity [112]. Splicing allows a single gene to encode functionally distinct protein isoforms. However, it can also cause protein malfunctioning and even a complete loss of protein production, particularly in relation to genetic disorders [113]. At this point, spatiotemporal regulation of splicing gains particular prominence. Herein, photoswitchable RNA binders enable spatiotemporal regulation splicing at the mRNA and translational level in living systems. Different RNA binding capacities of the isomers of **32** pave the way for assessment of SMN2 exon 7 inclusion activity through a luciferase reporter gene‐based assay in HEK293T cells (Figure 18). Thus demonstrating that the photocontrol of mRNA levels is possible through a photoswitchable RNA binder.

A ternary supramolecular complex system made up of azobenzene–spermine conjugate and CD that is reversibly able to capture and release single‐stranded DNA through photoisomerization by irradiation with visible light (455 nm) and *UV*‐light (355 nm) [114]. When the azobenzene moiety is in the *E*‐state, a ternary complex with 50‐mer single‐stranded DNA forms through strong non‐covalent, multivalent interactions. The *E*/*Z*‐photoisomerization multivalent interactions are disrupted to monovalent, low‐affinity interactions, inducing dissociation of the DNA. Similar reversible capture and release performance could not be achieved for double‐stranded DNA with 2000 base pairs. The represented complex, equipped with suitable targeting units, offers a promising approach for the controlled delivery of low molecular weight DNA and RNA in gene therapy applications.

The introduction of molecules undergoing *E*/*Z*‐photoisomerization into biological systems enables the modulation of biological processes, such as gene expression, RNA splicing, epigenetic regulation, and DNA interactions, through the use of light. This approach also holds promise for translational applications such as gene therapy.

#### 2.2.3 Strategies Against Antibiotic Resistance Through Photoisomerization

**Figure 19:**
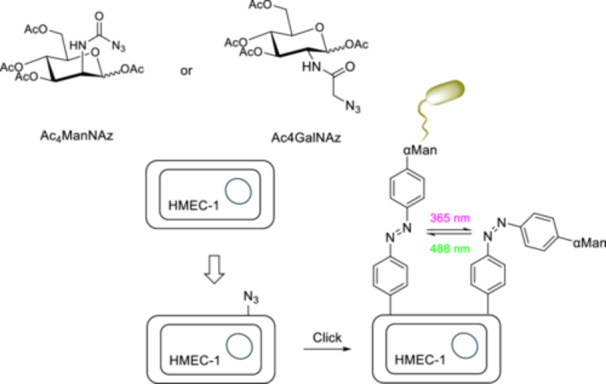
Schematic representation of azido labeling of HMEC‐1 by incorporating Ac_4_ManNAz into the terminal sialic acid units of membrane glycoproteins and Ac_4_GaINAz into mucin‐type glycoproteins and the conjugation of a‐d‐mannoside (αMan) bearing azobenzene *via* Click chemistry for the photocontrol regulation of bacterial adhesion to HMEC‐1.

Bacterial adhesion is a crucial process for bacterial cells, contributing to biofilm formation and bacterial colonization. The adhesion of bacterial cells to the host is also one of the initial processes of infection [115]. Thus, regulating bacterial adhesion is an effective alternative approach, allowing for the development of new methods for treating bacterial infections when taking bacterial resistance to antibiotics into consideration [116]. Bräuchle and Lindhorst et al. [117] employed azobenzene‐functionalized sugars to regulate the adhesion of *Escherichia coli* (*E*. *coli*) bacteria to human cells. Human microvascular endothelial cells, variant 1 (HMEC‐1) and GFP‐fluorescent type 1 fimbriated *E*. *coli* PKL116227 were used to study photocontrolled adhesion of bacterial cells to human cells. After incorporating Ac_4_ManNAz or Ac_4_GalNAz into HMEC‐1 for azido labeling, alkyne‐functionalized azobenzene glycosides were conjugated to the cell surface *via* Click chemistry (Figure 19).

High‐resolution live‐cell fluorescence microscopy was used to count the number of adhered bacterial cells after the interaction. Irradiation of the azobenzene derivative results in *Z* → *E* isomerization that enhances the number of bacterial cells adhered. The cell–cell adhesion was studied *via* flow‐based experiments and monitored by fluorescence microscopy of the sample irradiated with alternating 365–488 nm light cycles. The much higher slopes of the bacterial GFP signal were obtained upon irradiation at 488 nm until a certain number of cycles, after which HMEC‐1 were covered with bacterial cells.

**Figure 20:**
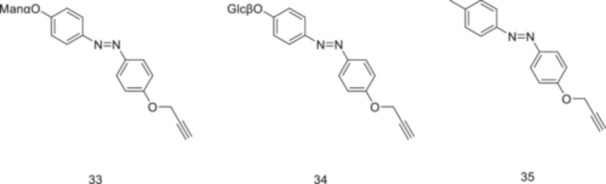
The α‐d‐mannopyranoside **33** is the principal structure to study cell–cell adhesion and β‐d‐glucopyranoside **34** and **35** are the control molecules.

Compound **34** was designed as a control compound due to its structural and polarity similarity to compound **33**, while lacking the bacterial targeting group. Compound **35** lacks a carbohydrate moiety entirely and also serves as a control compound. The adhesivity of bacterial cells was significantly decreased when interacting with control compounds **35** and **34**, which indicates that the adhesion of bacterial cells to HMEC‐1 in Figure 20 is carbohydrate‐specific.

Photoswitchable antibiotics, typically obtained by covalently attaching an antibiotic to a photoswitchable compound, hold promise as an alternative to address the growing challenge of antibiotic resistance [118]. A pronounced activity difference between isomers is essential for the effective application. Low or no antibacterial activity with stable isomer and high activity with metastable isomer should ideally be displayed [119]. In this context, various studies have been reported in the literature [120, 121, 122, 123]. One of the earlier studies on photoswitchable antibiotics reported [120] photoswitchable quinolones on quinolone‐sensitive *E*. *coli* CS1562 before and after irradiation at 365 nm and demonstrated a high antibacterial activity difference between the isomeric states. For the derivative with the largest difference in the activity, the non‐irradiated form exhibits a minimum inhibitory concentration (MIC) value equal to or larger than 64 µg/mL, while an MIC of 16 µg/mL is observed upon irradiation. The photomodulation of a new class of antibiotics, namely, cystobactamids, which are capable of inactivating bacterial DNA gyrase, modified by azobenzene, has been reported [122]. Antibiotic activity and gyrase inhibition were shown to be controllable by light. Moreover, a series of arylazopyrazole‐modified norfloxacin antibiotics were designed, and one of those derivatives exhibited an MIC of 0.25 μg/mL against norfloxacin‐resistant *Staphylococcus aureus* bacteria in a state formed upon irradiation, while the antibacterial efficiency dropped 24‐fold without irradiation [123].

Besides PDT with photoswitchable molecules as an alternative treatment of tumor tissues [50], the incorporation of *E*/*Z*‐photoswitches into antibacterial photodynamic therapy (aPDT) can elevate the aPDT, which is already an effective method toward antibacterial resistance [124], into a photocontrollable way [125, 126]. The development of antibiotic resistance by bacterial cells is occurring at a significant rate [127], and it is a challenging task to keep developing new classes of antibiotics. Light‐responsive bacterial inactivation strategies represent a promising alternative to conventional antibiotic therapies, especially in addressing the challenge of antibiotic resistance.

#### 2.2.4 Light‐Induced Modulation of Enzymatic Activity

The regulation of enzyme activities has also been addressed in the context of *E*/*Z*‐isomerizations (i.e., inhibition or enhancement of activities that are reversible). Through such strategies, biological activities can be potentially enabled and controlled through the incorporation of photoswitches. Hupfeld et al. [128] reported spatiotemporal control of enzyme activity using the photoswitchable property of azobenzene, particularly on two engineered variants of imidazole glycerol phosphate synthase complex (hW123 and fS55). The complex includes two subunits: a glutaminase (HisH) and a cyclase subunit (HisF). The azobenzene is located in the glutaminase subunit at the subunit interface, represented by hW123, or in the cyclase subunit, represented by fS55. The incorporation of azobenzene as an unnatural amino acid into the enzymes enables reversible control of enzyme activity upon monochromatic light irradiation. Interestingly, simultaneous dichromatic irradiation induces an alternative photocontrol mechanism through the formation of a second PSS, indicating the presence of a third transient species different from *E*‐ and *Z*‐isomers. Dichromatic irradiation provides reversible enhancement in the catalytic activity of the bienzyme complex imidazole glycerol phosphate synthase by affecting allosterically critical variant hW123 when varying between 365/420 nm irradiation and darkness. This study presents a promising approach for the selective control of different enzymes using distinct light modes. Another study on enzyme activity regulation focused on the development of an azobenzene‐modified nucleic acid probe [129]. Azobenzene‐modified DNA aptamers were utilized to regulate thrombin activity upon irradiation by wavelengths in the *UV*–vis region, as confirmed by measuring IC_200_ of different probe designs in clotting assays. The 15‐base‐long thrombin‐binding aptamer was selected as the inhibitory domain. Azobenzenes were incorporated into the complementary DNA sequence, and the two domains were linked by a polyethylene glycol linker. The spatial control of thrombin paves the way for site‐specific activation of coagulation, as demonstrated through studies in microfluidic channels. This approach offers potential tissue‐specific control of coagulation and inhibition of angiogenesis in cancer treatment. Light‐sensitive glycosidase regulators based on *p*‐ and *o*‐azobenzene α‐O‐glycosides of a 5*N*,6O‐oxomethylidene nojirimycin scaffold were designed [130]. The *p*‐octyloxy derivative among the designed molecules displays an exceptionally high switching factor (i.e., 1/2500 *E*‐to‐*Z*) for glycosidase inhibition. The most derivatives have *E*‐isomers that are more potent inhibitors, except for the *p*‐octyloxy derivative, which exhibits greater potency in the *Z*‐configuration. The study represents the first instance of reversible inhibition of human β‐glucocerebrosidase, exhibiting total ON–OFF switching behavior through *E*/*Z*‐photoisomerization of the *p*‐octyloxy derivative. The *Z*‐isomer of this derivative slowly reverts to the *E*‐isomer at physiological temperatures, allowing for “self‐deactivation” and offering potential as a pharmacological chaperone in Gaucher disease. Some derivatives in this study also function as “photocommutators,” enabling switching between α‐ and β‐glycosidase selectivity. Tan and Sun et al. [131] first reported the remote photocontrol of enzymatic activity in a confined environment. The study represents a strategy combining enzyme immobilization and photomodulation of immobilized enzyme activity. Carbonic anhydrase was employed as an enzyme to study the modulation of activity and a mesoporous photoresponsive inhibitor (PRI)‐decorated metal‐organic framework. The regulatory performance of PRI, a sulfanilamide‐substituted azobenzene motif, is significantly enhanced when it is in a confined space. The *E*‐configuration of PRI inhibits the enzymatic activity of immobilized carbonic anhydrase through noncovalent docking of the sulfanilamide group to the active site. Upon *UV*‐light irradiation, PRI reverts to the *Z*‐configuration, allowing active site of the enzyme to remain accessible.

Alternative methods to modulate enzyme activity include chemical inhibitors or cofactors [132, 133], pH or temperature changes [134, 135], and magnetic or electrical control [136, 137]. However, photomodulation of enzymatic activity offers an unprecedented approach for reversible spatiotemporal control of enzyme activities.

#### 2.2.5 Ion Channel Regulations and Mimicking Through Photoisomerization

The approaches toward optical control of ion channels and receptors, rendering them photocontrollable systems to elucidate neuroscientific processes through both chemical and genetic strategies, have been discussed before [138, 139, 140]. Herein, examples of *E*/*Z*‐photoisomerization for regulating ion channels and ion channel‐inspired ion transportation by photoswitchable systems are included, highlighting diverse molecular designs based on *E*/*Z*‐photoisomerization and their functional effects on synthetic ion transport and neural modulation.

An early example of ion channel blockage upon photoisomerization was reported in 1996 [141]. Azobenzene‐based molecular gates were designed to control gramicidin ion channels, which can transition between open and blocked states. One notable application of *E*/*Z*‐isomerization is the provision of allosteric control of the ionotropic glutamate receptor through a photo‐responsive agonist tethered to an engineered cysteine [142]. The azobenzene‐based system was designed to eliminate the requirement for a constant energy supply; the channel is OFF when it is in the *E*‐isomer, while azobenzenes thermally relax to a lower energy *E*‐state [143]. The same group disclosed the regulation of neurotransmission with high spatiotemporal precision using an azobenzene‐modified, glutamate‐based photochromic agonist, but non‐tethered for ionotropic glutamate receptors [144].

**Figure 21:**
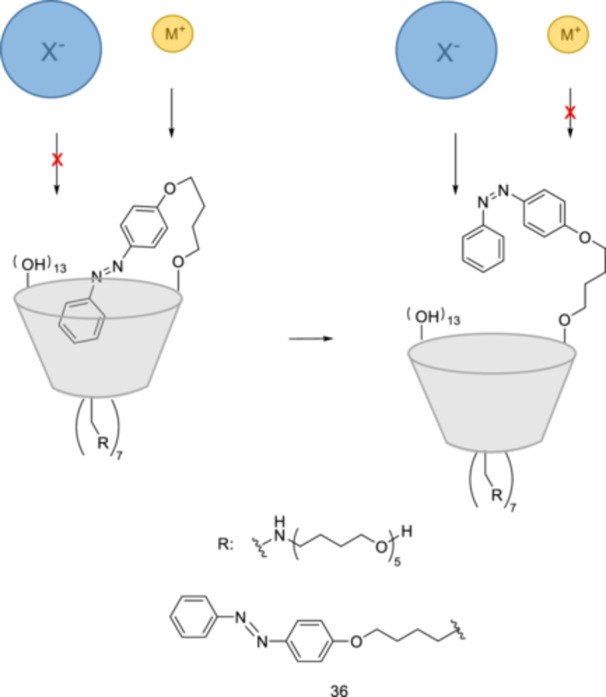
Schematic diagram of hypothesized anion and cation transport through the synthetic ion channels consisting of β‐CD and azobenzene derivative **36**.

Azobenzene‐modified gramicidin channels were regulated by light through isomerization. A light‐gated synthetic ion‐channel consisting of β‐CD and azobenzene as the pore and the gate, respectively, was designed [145]. The photoisomerization of the tethered‐*E*‐azobenzene **36** to *Z*‐azobenzene **36** leads to dissociation of the complex and faster anion and slower cation transport across a phospholipid vesicle membrane (Figure 21).

**Figure 22:**
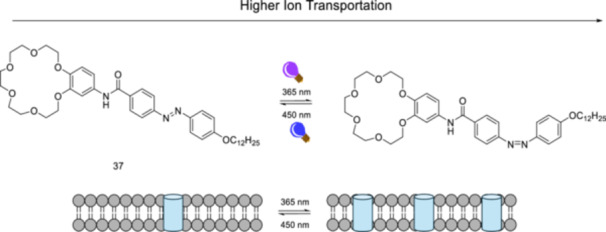
The regulation of ion transport by changing the number of opened gates upon photoisomerization of azobenzene‐based amphiphilic molecule **37**.

An azobenzene‐based amphiphilic small molecule **37** was reported to form light‐regulated ion channels [146]. *Z*/*E*‐isomerization of that building block **37** (Figure 22) results in the formation of two distinct states: *Z*‐ion channel and *E*‐ion channel, each demonstrating different transport characteristics. Photoisomerization of the ion channels leads to changes in the number of opened gates, thus regulating the transport of ions.

**Figure 23:**
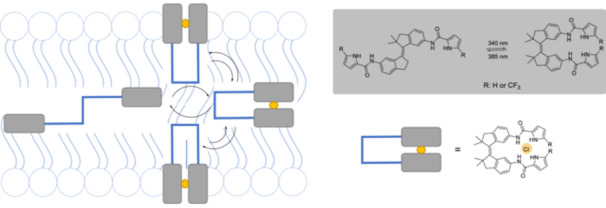
The modulation of the anion transport based on *E*‐ and *Z*‐isomers and photoisomerization of bis(amidopyrrole)‐functionalized stiff‐stilbene derivatives.

Two bis(amidopyrrole)‐functionalized stiff‐stilbene derivatives (Figure 23), having dimethyl‐substituted five‐membered rings, were synthesized that can undergo *E*/*Z*‐photoisomerization [147]. Interestingly, the isomers show different chloride transport activities although exhibiting similar anion binding affinities.

The regulation of anion transport is further showcased by an *E*‐azobenzene‐based photoregulatory anionophoric system [148], transporting chloride ions across the lipid membrane by forming a sandwich dimeric complex. *E*/*Z*‐isomerization upon irradiation at 365 nm weakens anion binding and transport due to structural changes, and ion transport ability is restored by irradiation at 450 nm through back‐isomerization. A light‐regulated synthetic ion channel study based on an azobenzene‐substituted tri(macrocycle) hydraphile was reported in 2015 [149]. The transmembrane activity of the hydraphile can be regulated upon irradiation with varying wavelengths, ranging from 365 nm *UV*‐light irradiation to 450 nm visible light irradiation, demonstrating a potential strategy for obtaining photocontrolled ion channels by regulating the transmembrane length of molecules with higher transmembrane activity switching.

Recently, light‐induced *E*/*Z*‐photoisomerization has expanded beyond artificial mimicry or control of ion channels to applications in direct neural modeling, activity imaging, and photopharmacological modulation of neural circuits. The first example of the use of an azobenzene‐based single near‐IR‐photon photoswitch for the actuation of metabotropic glutamate receptors in acute tissue slices was disclosed [150]. It allows non‐invasive modulation of neural signaling through deep tissue penetration. Near‐IR‐induced azobenzene photoswitches with two‐photon excitation properties and high thermal stability for the *Z*‐isomer demonstrated neuronal stimulation based on ionotropic glutamate receptors in both brain tissue and *Caenorhabditis elegans* nematodes, serving as models to analyze neuronal circuits [151]. The photoswitches with two‐photon excitation offer various advantages over one‐photon excitation, particularly in biological applications, including a longer wavelength of light with higher penetration depth, lesser phototoxicity, and the possibility of achieving high spatial resolution through the use of tightly focused beams [152]. These methodologies [150, 151] enable detailed molecular investigations of nervous system functions and support the development of novel therapeutic strategies.

#### 2.2.6 Photomodulation of Membrane Dynamics

The modulation of the cell membrane area is important for numerous cellular functions and structural adaptations. Cell division induces an increase in total cell surface area and requires the dynamic regulation of the cell membrane area [153]. Endocytosis causes a decrease in cell membrane area while enhancing cell membrane tension, and exocytosis exerts the opposite effect [154]. Cell membrane modulation is also involved in the signal transduction processes. For instance, endoplasmic reticulum‐plasma membrane junctions serve as platforms for initiating cell signaling cascades, requiring precise regulation of membrane composition and surface area [155]. The modulation of cell membrane areas is linked to the various diseases, including inflammatory [156] or neurodegenerative diseases such as Alzheimer's disease [157] and cancer [158]. Considering all these aspects, achieving precise spatiotemporal regulation of the cell membrane area is of importance. The photomodulation of cell membranes using photo‐sensitive molecules is an innovative and effective approach. The most abundant lipid component of the cell membranes is diacylglycerophospholipids [159], and the introduction of photoswitchable molecules into the phospholipids render them photo‐active. Two recent studies have reviewed the use of azobenzene‐ and hemithioindigo‐modified phospholipids, highlighting their applications in controlling membrane dynamics [160], as well as the use of azobenzene‐functionalized photolipids as molecular probes to investigate membrane properties and lateral organization [161].

The photomodulation of cell membrane area was achieved by intercalation of a photo‐active lipid, an amphiphilic azobenzene, into the plasma membranes [162]. *UV*‐light (365 nm) irradiation induces *E* → *Z* isomerization of the amphiphilic azobenzene, while irradiation at 490 nm promotes *Z* → *E* photoisomerization. The *E*‐isomer exhibited a higher affinity toward the red blood cell plasma membrane and better intercalation, thereby inducing a crenated echinocyte shape [163] in red blood cell membranes. The *Z*‐isomer is excluded from the plasma membrane, resulting in a discocyte‐like shape [163] for the membrane and a decrease in total membrane surface area. Variations in affinity between the photoswitching states of amphiphilic azobenzene for the plasma membrane enable reversible and rapid photomodulation of the cell membrane area.

**Figure 24:**
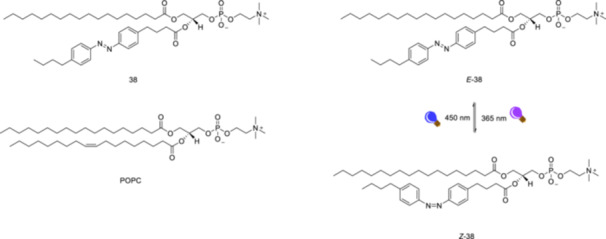
The structure of photolipid, azobenzene‐phosphatidylcholine **38** and phospholipid palmitoyl‐oleoyl phosphatidylcholine (POPC) and *E*/*Z*‐isomerization of the photolipid.

A recent study [164] demonstrated the use of the photo‐active lipid azobenzene‐phosphatidylcholine **38** (Figure 24) to modulate membrane physical properties using light. Membranes doped with azobenzene‐phosphatidylcholine **38** exhibited increased membrane area and reduced bending rigidity as a result of *E*/*Z*‐isomerization of the photo‐active lipid. *E*‐**38** bilayers exhibit greater specific membrane capacitance and dielectric constant compared with bilayers composed of palmitoyl‐oleoyl phosphatidylcholine (POPC), indicating that *E*‐**38** bilayers have a higher ability to store electric charges across the membrane.

According to Monari et al. [165], a cyclocurcumin derivative is a potential chemotherapeutic agent and offers an alternative approach for PDT with a reactive oxygen species generation independent pathway. Photoswitchable molecules, such as the cyclocurcumin derivative **39**, induce perturbation of lipid membranes through *E*/*Z*‐isomerization, offering a potential treatment for hypoxic solid tumors.

In a previous study by the same group [166], a pure 1,2‐dipalmitoyl‐phosphatidylcholine (DPPC) lipid bilayer was simulated, and only *E*‐**39** was calculated to represent the absolute minimum inside the membrane and at a position close to the polar head groups, and *Z*‐**39** represented only the metastable state in the lipid membrane.

**Figure 25:**
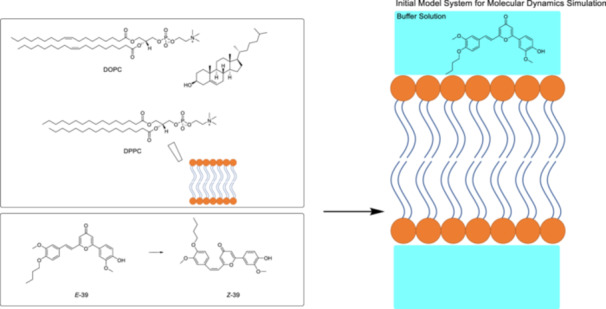
Neutral lipid components comprising bilayer system with molar ratio of DOPC:DPPC:Cholesterol 75:20:5 mimicking eukaryotic outer cell membrane and *E* and *Z*‐isomers of the cyclocurcumin derivative **39** (left). The initial model system consisted of a cell membrane model and a buffer solution containing NaCl at physiological concentration, and the cyclocurcumin (right).

The eukaryotic outer cell membrane model system, consisting of dioleoylphosphatidylcholine (DOPC), DPPC, and cholesterol (Figure 25) lipids was simulated by all‐atom molecular dynamics (MD). The membrane model was surrounded by a water buffer containing NaCl at a physiological concentration of 0.15 M and the cyclocurcumin derivative **39**. According to MD simulations, in the present study [165], both isomers spontaneously penetrate inside the membrane and are also located in the vicinity of the head groups, contrary to the previous study [164]. Notably, it was demonstrated that *Z*‐**39** enlarges the area per lipid more than *E*‐**39**, indicating a stronger membrane disruption. However, the perturbation of the membrane was less pronounced compared to the single‐lipid membrane. According to Monari et al. [165], more bulky and rigid peripheral substituents are required to enhance membrane perturbation capability upon photoisomerization.

Membrane mobility or fluidity affects various membrane‐dependent biological processes such as ion transport, signal transduction, and protein function [167, 168]. The study by Wezenberg et al. [169] represents a strategy to modulate membrane mobility and transmembrane transport activity. Synthetic transmembrane anion transporter equipped with azobenzene photoswitches were designed to modulate the transport activity by changing the incorporation of the transporter and thus membrane mobility. The thermodynamically stable PSS of the functionalized transporter shows incorporation into the bilayer to a lesser extent and *E*/*Z*‐isomerization by *UV*‐light irradiation enhances the incorporation and decreases membrane mobility and transport activity.

Pritzl and Lohmüller et al. [170] investigated the vesicle fusion of small unilamellar photo‐active lipid vesicles into the giant unilamellar vesicles for the optical control of membrane properties. Two different fusion strategies were tested, including photoinduced and charge‐mediated fusions. Charge‐mediated fusion using ionic lipids is more effective than photoinduced fusion employing neutral phospholipids, in terms of doping efficiency. A mono‐azobenzene moiety bearing a phosphatidylcholine analog enables reversible control of the membrane order and phase separation, while a phosphatidylcholine analog with two azobenzene moieties is effective on membrane fluctuations and vesicle shape of the giant unilamellar vesicles after fusion. To enhance the compatibility of the photo‐active lipid in vivo studies, an azobenzene‐based red‐shifted photo‐active lipid was designed and incorporated into giant unilamellar vesicles through charge‐mediated fusion, enabling the photocontrol of vesicle stiffness.

Murphy et al. [171] employed photoswitchable azobenzene glycolipids incorporated into DPPC Langmuir monolayers to obtain a model system for further investigation of membrane structure–function relationships. *E*/*Z*‐photoisomerization of azobenzene glycolipids, upon 455 nm to 365 nm irradiation, induces structural changes in DPPC monolayers through monolayer reorientation with a crossover point. Above this point, the reorientation behavior of the molecules exhibits opposite trends between pressure and layer thickness, indicating that embedded azobenzene glycolipids change their orientation. While *E*/*Z*‐photoisomerization of the azobenzene glycolipids increases the occupied area by the glycolipids with elevated surface pressure below the point.

**Figure 26:**
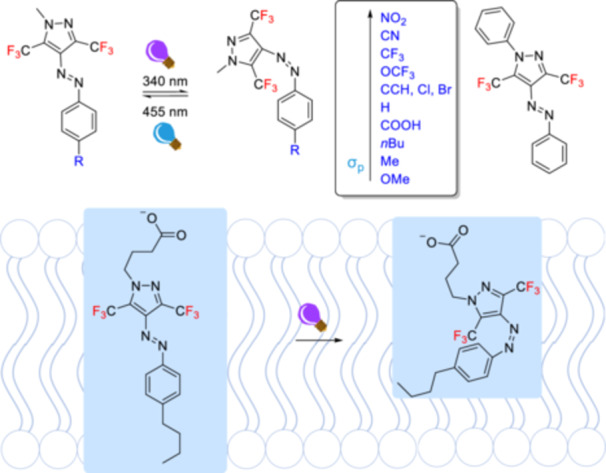
The structures and photoisomerization of phenylazopyrazole derivatives (σₚ= The Hammett para‐substituent parameter) and phenylazopyrazole fatty acid that is incorporated into a lipid bilayer.

Arylazopyrazoles are promising candidates as photoswitches with high photoisomerization quantum yields, relatively high thermal stability of metastable *Z*‐isomers, and quantitative reversibility between the states [172]. Different hexafluoroarylazopyrazoles have been previously designed but have not been considered as ^19^F probes [173, 174]. A very recent study demonstrates the synthesis and photoisomerization of a series of differently substituted arylazopyrazoles bearing two trifluoromethyl groups as potential photoswitchable ^19^F probes to circumvent the limitations of routine analytical techniques in complex systems such as membranes or biomimetic systems [175]. Although fluorinated arylazopyrazoles developed in this study exhibit slightly lower photoisomerization quantum yields and photostationary distributions for *E*/*Z* photoisomerization than non‐fluorinated ones, and cannot quantitatively revert to the stable isomer, the recovery percentage of *E*‐isomers ranges from 70% to 84% depending on the substituents. The λ_max_ value of the nπ* transition was observed to vary with the Hammett‐Taft parameters [176]. Moreover, a reverse trend was observed between the electron‐donating character of the substituents and the photoisomerization quantum yields of the arylazopyrazoles (Figure 26). The arylazopyrazole fatty acid was incorporated into a vesicle with a lipid composition of the fatty acid, DOPC, and cholesterol in a 15/32.5/2.5 (w/w/w) ratio, and photoisomerization of the fatty acid was observed *via* ^19^F NMR.

It is possible to alter membrane dynamics by modifying lipid composition [177], cholesterol content [178], or membrane protein expression [179]. However, photomodulation of membrane dynamics through *E*/*Z*‐photoisomerization not only enables the regulation of the structural properties of membranes but also their functions, offering control over membrane‐associated biological processes and properties, such as signaling and permeability, in a reversible manner.

#### 2.2.7 Photocontrolled Drug Delivery Systems Through Isomerization

Drug delivery systems are designed to deliver the drugs in a controlled and targeted manner to enhance therapeutic effects and minimize side effects [180]. The most commonly used drug delivery systems are composed of nanostructures, including liposomes and micelles [181]. The incorporation of photoswitchable molecules into drug delivery systems enables the non‐invasive control of drug delivery, depending on the wavelength of the irradiation. Doxorubicin was loaded into the mesopores of azo‐modified, mesoporous silica‐coated upconverting nanoparticles [182]. Near‐IR light at 980 nm absorbed by the nanoparticles induces *UV*‐vis emission, which provides the energy for the reversible *E*/*Z*‐photoisomerization of the azobenzene moiety. This simultaneous *UV*‐vis emission drives continuous photoisomerization, creating a wagging motion that acts as a molecular impeller to release doxorubicin.

A novel chemiluminescence‐based strategy for target‐specific, controlled drug delivery by tissue depth‐independent photoisomerization has been developed [183]. Although self‐luminescent systems are independent of tissue depth, they generally produce low‐intensity light, making photoisomerization inefficient [184]. Chemiluminescence substrate, peroxyoxalate, and chemiluminescence fluorophore antitumor drug camptothecin were co‐encapsulated by host–guest nanoparticles composed of azobenzene‐pendant polymer and a cyclodextrin‐pendent polymer. H_2_O_2_‐induced chemiluminescence triggers the photoisomerization of the azobenzene moiety, leading to the release of camptothecin. High levels of H_2_O_2_ are associated with various biological problems, including the development of cancer cells [185], inflammation in tissues [186], and neurodegenerative disorders [187].

**Figure 27:**
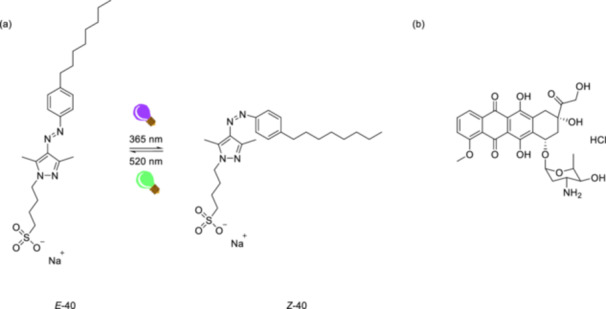
Photoisomerization of arylazopyrazole surfactant **40** (a) and the structure of doxorubicin (b).

The study by Braunschweig et al. [188] reports photo‐induced drug release using a micellar nanocarrier based on arylazopyrazole surfactant **40** at the air–water interface. The air–water interfaces serve as models for the organic‐aqueous interface of cells [189], and the interactions between drugs and carriers are important at these interfaces for drug delivery [190]. Photoisomerization of the arylazopyrazole surfactants alters CMC values; *Z*‐**40** possesses a significantly higher CMC value than *E*‐**40** (Figure 27). *E*/*Z*‐photoisomerization induces the release of doxorubicin encapsulated in the micellar structure of *E*‐**40** at the interface.

Nanocomposites of phosphatidylcholine vesicles and azobenzene nanoclusters were formed to obtain a photoresponsive drug delivery system [191]. Similarly, doxorubicin was loaded into the nanocomposites with enhanced loading capacity for phosphatidylcholine‐azobenzene nanocomposite vesicles compared with phosphatidylcholine vesicles. Azobenzene‐containing vesicles also exhibited enhanced drug release rates upon irradiation at 365 nm at different pH values.

Drug delivery systems are employed for the targeted delivery of the drugs to enhance their activities while minimizing possible side effects. The incorporation of biocompatible photoswitches that switch with light, possessing suitable penetration depths and low phototoxicity, into drug delivery systems enables the controlled release of drugs in a non‐invasive manner.

## 3 Conclusion and Outlook

It has been highlighted how supramolecular assembly with small molecules that can undergo *E*/*Z*‐isomerizations impacts the reaction behavior. Different reaction outcomes can be observed upon assembly when compared with the free molecules. Changes in the microenvironment surrounding the molecule cause distinct differences in photophysical properties and the resulting reaction outcome. For example, restricting the freedom of movement for small molecules in assembly can increase emissions. Such changes caused by assembly can be applied in a biological context. Hence, lessons learned from supramolecular assemblies that increase our understanding of the effects of local environments and interactions have the potential to be translated into biological applications. Biological applications related to fluorescence, regulation, and modifications of genes, bacteria, ion channels, neural systems, enzymes, cell membrane dynamics, and drug delivery systems have been showcased to illustrate the importance of *E*/*Z*‐isomerizations in biomimetic and biological contexts.

While several groups are actively employing *E*/*Z*‐isomerizations as a powerful tool in biological contexts, there is potential for further developments and applications. It is important in this context to invest efforts not only in characterizing the photophysical properties and reaction behavior in organic solvents and water but also in more complex biomimetic media. Suitable biomimetic media can be represented by membranes or hosts that mimic the microenvironment of the intended application. We hope that this review supports further interest and developments to increase the understanding of photochemical reaction behavior in more complex microenvironments.

## Conflicts of Interest

The authors declare no conflicts of interest.
